# Integrated analysis of SKA1-related ceRNA network and SKA1 immunoassays in HCC: A study based on bioinformatic

**DOI:** 10.1097/MD.0000000000034826

**Published:** 2023-09-22

**Authors:** Fanjing Zeng, Zhiqi Xu, Peng Zhuang

**Affiliations:** a Department of Infectious Disease, The Eighth Affiliated Hospital of Sun Yat-sen University, Shenzhen City, China.

**Keywords:** bioinformatics, competing endogenous RNA, GSEA, hepatocellular carcinoma, immune, prognosis, SKA1

## Abstract

Hepatocellular carcinoma (HCC) poses a global health challenge. Effective biomarkers are required for early diagnosis to improve survival rates of patients with HCC. Spindle and kinetochore-associated complex subunits 1 (SKA1) is essential for proper chromosome segregation in the mitotic cell cycle. Previous studies have shown that overexpression of SKA1 is associated with a poor prognosis in various cancers. The expression, prognostic value, and clinical functions of SKA1 in HCC were evaluated with several bioinformatics web portals. Additionally, we identified target long non-coding RNAs (lncRNAs) and microRNAs by analyzing messenger RNA (mRNA)-miRNA and miRNA-lncRNA interaction data and elucidated the potential competing endogenous RNA (ceRNA) mechanism associated with SKA1. High SKA1 expression was associated with poor prognosis in patients with HCC. Furthermore, multivariate Cox regression analysis revealed that SKA1 expression was an independent prognostic factor for HCC. GO and KEGG analyses showed that SKA1 is related to the cell cycle checkpoints, DNA replication and repair, Rho GTPases signaling, mitotic prometaphase, and kinesins. Gene set enrichment analysis revealed that high levels of SKA1 are associated with cancer-promoting pathways. DNA methylation of SKA1 in HCC tissues was lower than that in normal tissues. Ultimately, the following 9 potential ceRNA-based pathways targeting SKA1 were identified: lncRNA: AC026401.3, Small Nucleolar RNA Host Gene 3 (SNHG3), and AC124798.1-miR-139-5p-SKA1; lncRNA: AC26356.1, Small Nucleolar RNA Host Gene 16 (SNHG16), and FGD5 Antisense RNA 1-miR-22-3p-SKA1; lncRNA: Cytoskeleton Regulator RNA (CYTOR), MIR4435-2 Host Gene, and differentiation antagonizing non-protein coding RNA-miR-125b-5p-SKA1. SKA1 expression levels significantly correlated with immune cell infiltration and immune checkpoint genes in the HCC tissues. SKA1 is a potential prognostic biomarker for HCC. This study provides a meaningful direction for research on SKA1-related mechanisms, which will be beneficial for future research on HCC-related molecular biological therapies and targeted immunotherapy.

## 1. Introduction

Liver cancer is the fourth leading cause of cancer mortality.^[1]^ The 5-year relative survival rate is approximately 18%. By 2030, the annual liver cancer fatalities are expected to surpass 1 million.^[1]^ Hepatocellular carcinoma (HCC) is the most prevalent type of liver cancer and accounts for 90% of all cases. HBV infection is the most common risk factor for HCC development, accounting for 50% of HCC cases.^[2]^ The highest incidence rates of HCC have been observed in Asia and Africa,^[3]^ where chronic hepatitis B infection is the predominant risk factor and exposure to chronic hepatitis B is the main risk factor.^[4]^ Over the past 2 decades, there has been minimal progress in the identification of clinically effective biomarkers for early diagnosis of HCC.^[5]^ The only blood test currently available for the noninvasive detection of HCC is alpha-fetoprotein (AFP); however, its clinical applicability is restricted owing to its poor sensitivity and specificity.^[6]^ Therefore, reliable diagnostic biomarkers are urgently required to identify early stage HCC.

Long non-coding RNAs (lncRNAs) are a subtype of ncRNAs with a length larger than 200 nt but no or restricted protein-coding capacity.^[7]^ According to earlier studies on non-coding RNAs, many lncRNAs control gene expression during or after transcription processes. lncRNAs influence a variety of pathological and physiological processes via biological control, including chromosomal imprinting, epigenetic regulation, cell proliferation, and cell cycle progression.^[8,9]^ MicroRNAs (miRNAs), which are short single-stranded ncRNAs with 19-25 nucleotides (nt), may attach to the 3′ untranslated region of their target mRNA to prevent gene expression or translation.^[10–12]^ According to the newly developed potential ceRNA hypothesis, lncRNAs compete for a large number of miRNAs in the cell and function as sponges, buffers, and interferes with the protein produced by the target gene mRNA.^[13]^ Nevertheless, research on Hepatocellular carcinoma is scarce, and there is still a need for a full investigation of the lncRNA-miRNA-mRNA ceRNA regulation network connected to HCC using high-throughput sequencing and a large sample size.

**Figure 1.**
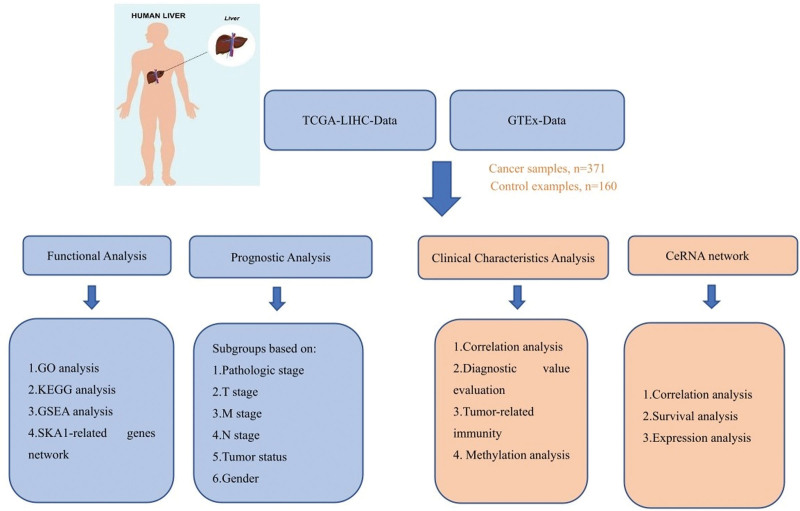
An overall flowchart of this work.

Spindle and kinetochore-associated complex subunit 1 (SKA1) is an outer kinetochore microtubule-binding protein that is required for kinetochore-spindle microtubule attachment and correct chromosomal segregation during mitosis.^[14]^ SKA1 protein deficiency may lead to serious chromosomal separation abnormalities.^[15]^ Many studies have demonstrated a link between high SKA1 expression and poor prognosis in cancer. Wang et al^[16]^ discovered that elevated SKA1 expression in glioma patients was strongly associated with tumor stage and poor prognosis. SKA1 has also been shown to be increased in prostatic tumor tissue, influencing vascular lymphatic invasion, metastasis, and tumor stage.^[17]^ Moreover, higher SKA1 expression was shown to be substantially associated with tumor size and degree of cellular differentiation in pancreatic ductal adenocarcinoma.^[18]^ According to large peptide sequencing data from the HUMAN PROTEOME MAP project, SKA1 demonstrates a high expression level in the fetal liver with vigorous cell division, then progressively decreases throughout development, and remains at a very low level in the adult liver.^[19]^ These pathways play a significant role in the incidence and progression of liver cancer. There is still much potential for investigation into the regulatory mechanism upstream of SKA1. It is possible to prevent HCC development by determining the most effective regulatory mechanism associated with SKA1 and suppressing its expression. We aimed to investigate the mechanism and associated regulation of SKA1 in HCC as our present knowledge is limited. Consequently, we used bioinformatics to examine the lncRNAs and miRNAs associated with SKA1 and investigated the relationship between SKA1 and immune infiltration levels of different types of immune cells in HCC to discover possible biological targets and prepare for future fundamental research. The Declaration of Helsinki served as the foundation for this investigation (revised in 2013). The study was divided into 2 parts. First, we used TCGA database to confirm that SKA1 was highly overexpressed in several malignant tumors, including HCC. Kaplan–Meier (K–M) survival curve analysis and time-”ependent ROC curve analysis were used to evaluate SKA1’s prognostic and diagnostic value in hepatocellular carcinoma. Second, we grouped 373 instances of hepatocellular carcinoma into distinct subgroups according to clinical and pathological criteria and conducted subgroup K–M survival curve analysis to further analyze the association between SKA1 expression and survival. Third, we performed bioinformatics analysis to identify putative ceRNA processes associated with SKA1, and we identified 9 potential signaling pathways. We then employed the Tumor Immune Estimate Resource (TIMER) database to undertake an immune infiltration study and investigate the link between SKA1 and tumor-related immunity. A flowchart of this study is presented in Figure 1. To determine SKA1’s diagnostic and prognostic values in HCC, Cox regression analysis was performed. Gene ontology (GO) and Kyoto Encyclopedia of Genes and Genomes (KEGG) analyses were used to determine potential role of SKA1 in HCC. Finally, methylation analysis was carried out to examine SKA1 DNA methylation levels and its prognostic value in HCC.

## 2. Methods

### 2.1. Data collection, ROC curves, and prognostic survival curve of SKA1

The TCGA database (https://portal.gdc.cancer.gov/) was used to download and arrange RNA-seq data from TCGA-LIHC experiments and patient clinical data. Predictive information was obtained from a previous cell publication.^[20]^ The TIMER2.0 database (http://timer.cistrome.org/) was used to obtain a differential expression map of SKA1 in all cancer types. The “limma,” “stats,” and “car” packages were used to evaluate the differential expression of SKA1 in HCC. The “timeROC” software was used to perform the time-dependent ROC analysis. Using the “survival” package, the proportionate risk hypothesis test and fitting survival regression were performed. Displays the outcomes of the “ggplot2” and “survminer” programmes.

### 2.2. Differentially expressed gene analysis

We used the unpaired Student *t* test within the DESeq2 R package (3.6.3)^[21]^ to compare the expression data (Htseq-counts) between the high- and low-expression groups, according to the median SKA1 expression level. The thresholds for the differentially expressed gene (DEGs) were |log2-fold change (FC)| > 1.5 and adjusted *P* < .05.

### 2.3. Enrichment analysis

Protein-protein interaction network analysis was performed using the BioGRID website.^[22]^ The top 100 SKA1-correlated genes from TCGA-LIHC tumor and normal tissues were obtained and included in the GEPIA2.0 database. We then used pairwise gene-gene Pearson correlation analysis to determine the relationship between SKA1 and the chosen genes. We examined the underlying biological processes and signaling pathways affected by SKA1 in TCGA-LIHC tumors using GO and KEGG enrichment analyses. Statistical significance was set at *P* < .05. Based on transcriptional sequences from TCGA data, we used gene set enrichment analysis (GSEA) to explore gene sets and pathways related to SKA1. Gene expression data used in this study were divided into SKA1 groups with high and low expression levels. We used the R package cluster profiler to run GSEA on the Broad Institute website to compare the 2 groups and identify the probable functions.^[23,24]^

### 2.4. Subgroup K–M prognostic survival analyses of SKA1

The TCGA database (https://portal.gdc.cancer.gov) was used to obtain and arrange RNA-seq data and clinical information for TCGA-LIHC experiments. RNA-seq data were then extracted in TPM format. A total of 373 patients with HCC were divided into subgroups based on clinical and pathological factors. Using the Kaplan–Meier survival “survival” package, the proportional risk hypothesis test and fitting survival regression were performed, and the results were then shown using the “survminer” and “ggplot2” tools.

### 2.5. Correlation analysis between SKA1 expression levels and clinicopathological characteristics of hepatocellular carcinoma patients

Overall survival (OS), disease-specific survival (DSS), and progression-free interval (PFI) data for HCC patients were obtained from the TCGA-LIHC project and from a previously published analysis of HCC patients.^[20]^ The R program was used to examine the differences between the high- and low-EXO1 expression groups in several clinicopathological characteristics, including weight, AFP levels, DSS events, OS events, T stage, tumor status, histological grade, and pathological stage. The Shapiro–Wilk normality test (*P* < .05 for data with normal distribution), Kruskal–Wallis test, and Dunn’s Multiple Hypothesis Test were used to determine whether there were any differences between the groups. The Bonferroni correction was used to adjust the findings to a significant level. The “ggplot2” (v3.3.3) R software was used to display the statistical data. The correlation between SKA1 expression levels and clinicopathological characteristics of patients with HCC was assessed using logistic regression analysis.

### 2.6. Evaluation of the prognostic significance of SKA1 expression in hepatocellular carcinoma

The survival data of patients with HCC from TCGA–LIHC project and previously published data^[20]^ were examined using the K–M survival “survival” (v3.2-10) R package (statistical analysis) and “survminer” (v.0.4.9) R package (visualization) for prognostic analysis. K–M survival curve analysis and univariate and multivariate Cox regression analyses were used to estimate the survival of HCC patients depending on SKA1 expression levels. HCC subgroup prognosis was based on KM survival curves. The sample size (percentage), hazard ratio (HR), confidence interval (CI), and p-values are reported. The “ggplot2” (v3.3.3) R software was used to create forest plots.

### 2.7. Construction and validation of the nomogram

Multivariate Cox analysis was used to create a nomogram to predict overall survival. The discriminatory ability of the nomogram was measured using the concordance index and calibration plots. The R package RMS (version 5.1–4)^[8]^ was used to develop the nomogram and calibration graphs.

### 2.8. Methylation and expression analysis of SKA1

First, we used the Human Disease Methylation Database DiseaseMeth version 2.0, (http://bio-bigdata.hrbmu.edu.cn/diseasemeth/) and UALCAN (http://ualcan.path.uab.edu/) to assess the methylation levels of SKA1 between HCC and paracancerous normal tissues. The promoter methylation status in patients with various tumor grades, tumor stages, N stage, and age were also compared. We also investigated the association between hub gene expression and DNA methylation status by using MEXPRESS (https://mexpress.be).

### 2.9. Establishment of mRNA-miRNA-lncRNA interaction network and prognostic survival analysis of SKA1-related miRNA and lncRNA

The mRNA-miRNA and miRNA-lncRNA interaction data were downloaded from the starBase database (http://starbase.sysu.edu.cn/). A correlation coefficient (*R* value) > 2 or -2 (an *R* value > 2 was deemed favorably correlated, and an *R* value < −2 was considered negatively correlated), and a *P* value < .05 was used as a screening criterion. After examining the correlations between the data variables, the findings were analyzed using the “ggplot2” program. Differential expression and prognostic survival analyses were performed using the “survival” package and the “limma,” “stats,” and “car” packages, and the findings were shown using the “ggplot2,” “car” packages. BioRender was used to create a Conceptual Map Diagram of the SKA1-related ceRNA pathway in HCC (created using Biorender.com).

### 2.10. Correlation analyses of SKA1 with immune cells and immune checkpoints

The correlation analyses of SKA1 with various immune cells and several immune checkpoints were analyzed and visualized using various R language packages, including “limma,” “ggplot2,” “ggExtra,” “corrplot,” “reshape2,” “vioplot,” and “ggpubr.” The Spearman statistical method was used to calculate *P* values. An *R* value > 2 and a *P* value < .05 were considered to be positively correlated, an *R* value < −2 and a *P* value < .05 were considered to be negatively correlated, and a *P* value > .05 was considered to be not significant. SKA1, Programmed Cell Death 1, CD276 molecule, Lymphocyte Activating 3, Cytotoxic T-Lymphocyte Associated Protein 4, T Cell Immunoreceptor With Ig and ITIM Domains, TNF Superfamily Member 4, Lectin Galactoside-Binding Soluble 9, and TNF Receptor Superfamily Member 18 were analyzed using TIMER 2.0 database (http://timer.cistrome.org/).

### 2.11. Statistical analysis

R 3.6.3 was used to process statistical data obtained from TCGA. Wilcoxon rank-sum and Wilcoxon signed-rank tests were used to compare SKA1 expression levels between the HCC and control groups. Welch’s one-way analysis of variance was used to examine the relationship between SKA1 expression and the grade of clinicopathological variables, followed by the Bonferroni correction or *t* test. Univariate logistic regression, Fisher’s exact test, and normal and adjusted Pearson’s 2 tests were used to examine the influence of the clinicopathological variables on SKA1 expression. Both univariate and multivariate Cox regression analyses were used to assess the predictive significance of SKA1 expression and other clinicopathological variables for OS. Multivariate analysis included all factors from the univariate analysis. A K–M curve was created to investigate the predictive significance of SKA1. Univariate Cox proportional hazard regression was used to assess HR for OS, DSS, and PFI. Individual factor HR were calculated by measuring the HR with a 95% CI.

The pROC program was used to perform receiver operating characteristic (ROC) analysis of SKA1.^[25]^ The computed area under the curve value ranges of 0.5 to 1.0 showed 50% to 100% discriminating ability. SKA1 was evaluated for predicting HCC outcomes at 1, 3, and 5 years using time-dependent analysis of the ROC curve. All statistical tests were considered significant at 2-tailed *P* ≤ .05.

## 3. Results

### 3.1. Differential expression, ROC curves, and prognostic survival curve of SKA1

**Figure 2.**
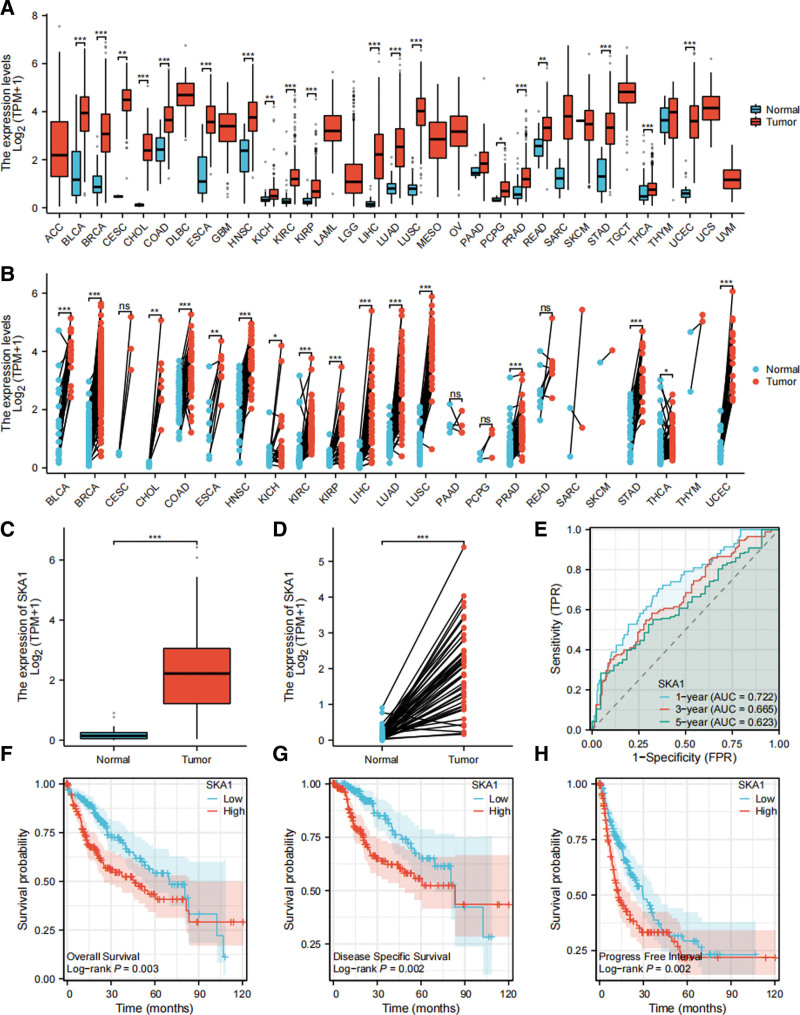
Differential expression, ROC curves and prognostic survival curve of SKA1. (A) Differential expression of SKA1 in HCC from the TIMER2.0 database; (B) pairwise differential expression analysis of SKA1 in HCC from the TIMER2.0 database; (C) differential expression of SKA1 between the tumor group and the normal group in HCC from TCGA-LIHC database; (D) pairwise differential analysis of SKA1 in HCC (*P < .05; **P < .01; ***P < .001); (E) time-dependent ROC curves analysis from TCGA-LIHC database; (F) Overall survival curve from the GEPIA database; (G) disease-free survival curve from the GEPIA database; and (H) progress free interval curve from the GEPIA database. GEPIA = gene expression profiling interactive analysis, HCC = hepatocellular carcinoma, LIHC = liver hepatocellular carcinomas, ROC = receiver operating characteristic, TCGA = The Cancer Genome Atlas, TIMER = Tumor IMmune Estimation Resource.

In the TIMER2.0 database, we examined the expression of SKA1 in 38 cancer types and discovered that SKA1 was substantially differentially expressed in the tumor group in numerous malignant tumor forms, including HCC (*P* < .001; Fig. 2A and B). We downloaded gene expression data and related clinical information data from the TCGA-LIHC database for patients with HCC and discovered that SKA1 was substantially differentially expressed in malignant tumors (*P* < .001; Fig. 2C and D). In addition, we conducted a time-dependent ROC analysis to evaluate the prognostic value of SKA1. After 1, 3, and 5 years, the area under the curve for the prognostic signature were 0.722, 0.665, and 0.623, respectively (Fig. 2E). The time-dependent ROC curve revealed that SKA1 had positive predictive value. Subsequently, we performed a prognostic survival analysis of SKA1-related OS, disease-free survival (DFS), and PFI of HCC patients in the GEPIA database. The prognostic survival curves indicated that the prognosis of the high-risk group (186 patients) differed significantly from that of the low-risk group (187 cases) (OS: *P* = .002; DFS: *P* = .003; PFI: *P* = .003). Compared to patients in the high-risk group, those in the low-risk group had notably better prognosis in terms of OS (Fig. 2F), DFS (Fig. 2G), and PFI (Fig. 2H).

### 3.2. Identification of differentially expressed genes in hepatocellular carcinoma

**Figure 3.**
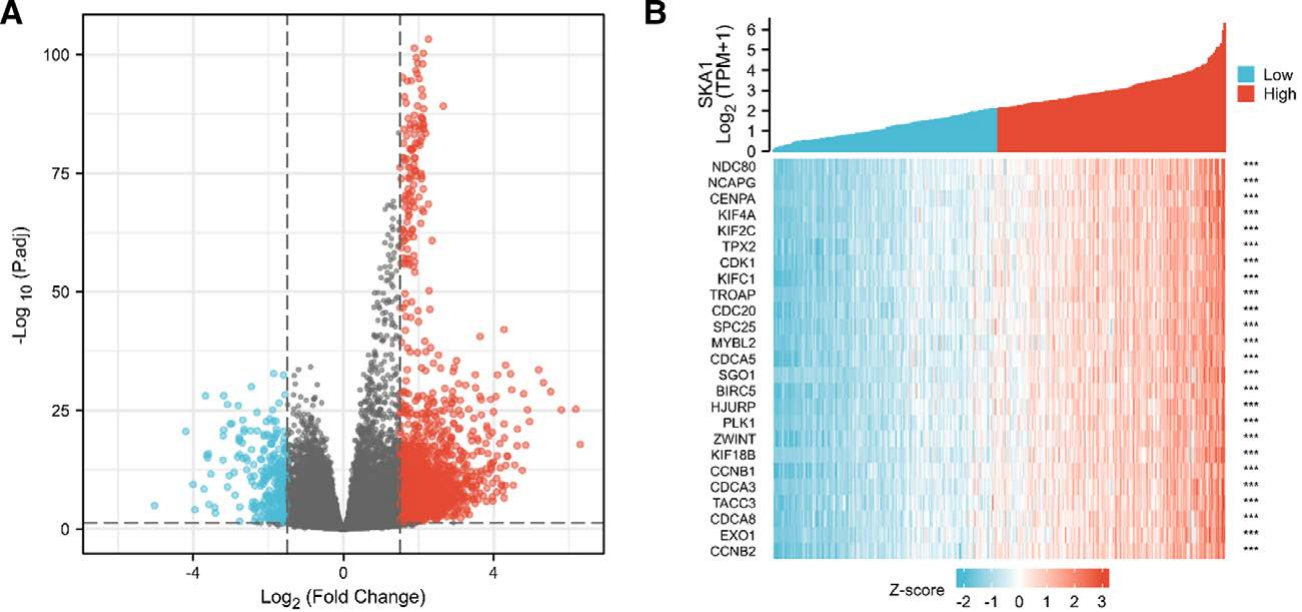
Results of DEG analysis. (A) Volcano plot of differentially expressed RNAs. (B) Heat map of the 25 differentially expressed genes. DEG = differentially expressed gene.

Using |logFC| <1.5 and adjusted *P* < .05, as the cutoff criterion, a total of 1880 DEGs (1606 upregulated and 274 downregulated) were identified by examining TCGA HTSeq-Counts data of SKA1-related genes. The expression of DEGs was represented by a volcanic plot (Fig. 3A). A heat map was created to show the relationship between SKA1 and the 25 genes (Fig. 3B).

### 3.3. Functional enrichment analysis of SKA1

**Figure 4.**
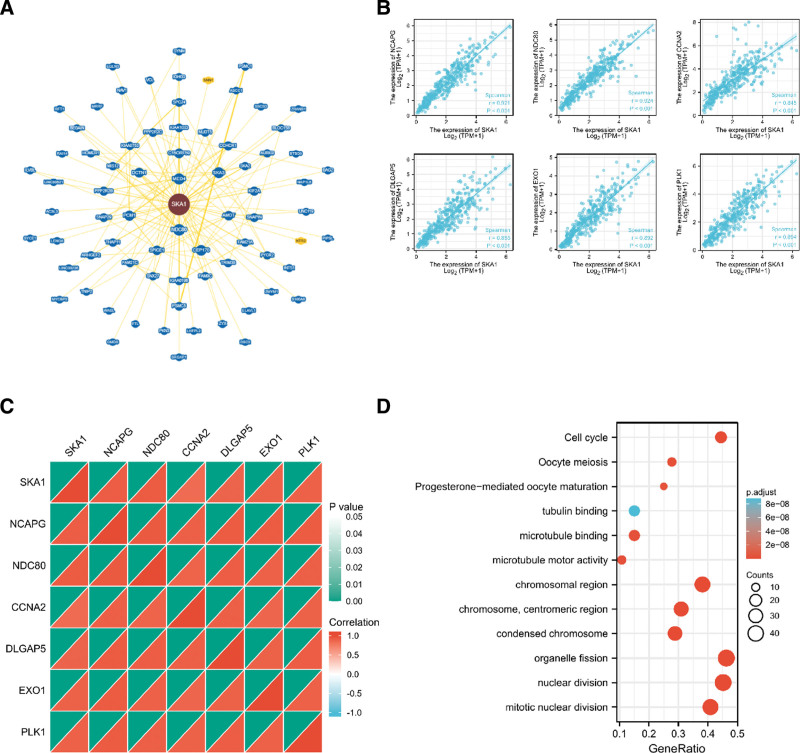
Functional enrichment analysis of SKA1-related genes. (A) SKA1-related genes were obtained from the BioGRID web tool, and 73 proteins were displayed. (B) GEPIA2.0 showed the positive correlations between SKA1 and 6 genes (NCAPG, NDC80, CCNA2, DLGAP5, EXO1, and PLK1). *P* value < .001. (C) The heatmap confirmed that SKA1 expression was positively correlated with the 6 genes (NCAPG, NDC80, CCNA2, DLGAP5, EXO1, and PLK1) in HCC. (D) GO and KEGG enrichment analyses of SKA1-related genes. GO = Gene Ontology, HCC = hepatocellular carcinoma, KEGG = Kyoto Encyclopedia of Genes and Genomes.

**Figure 5.**
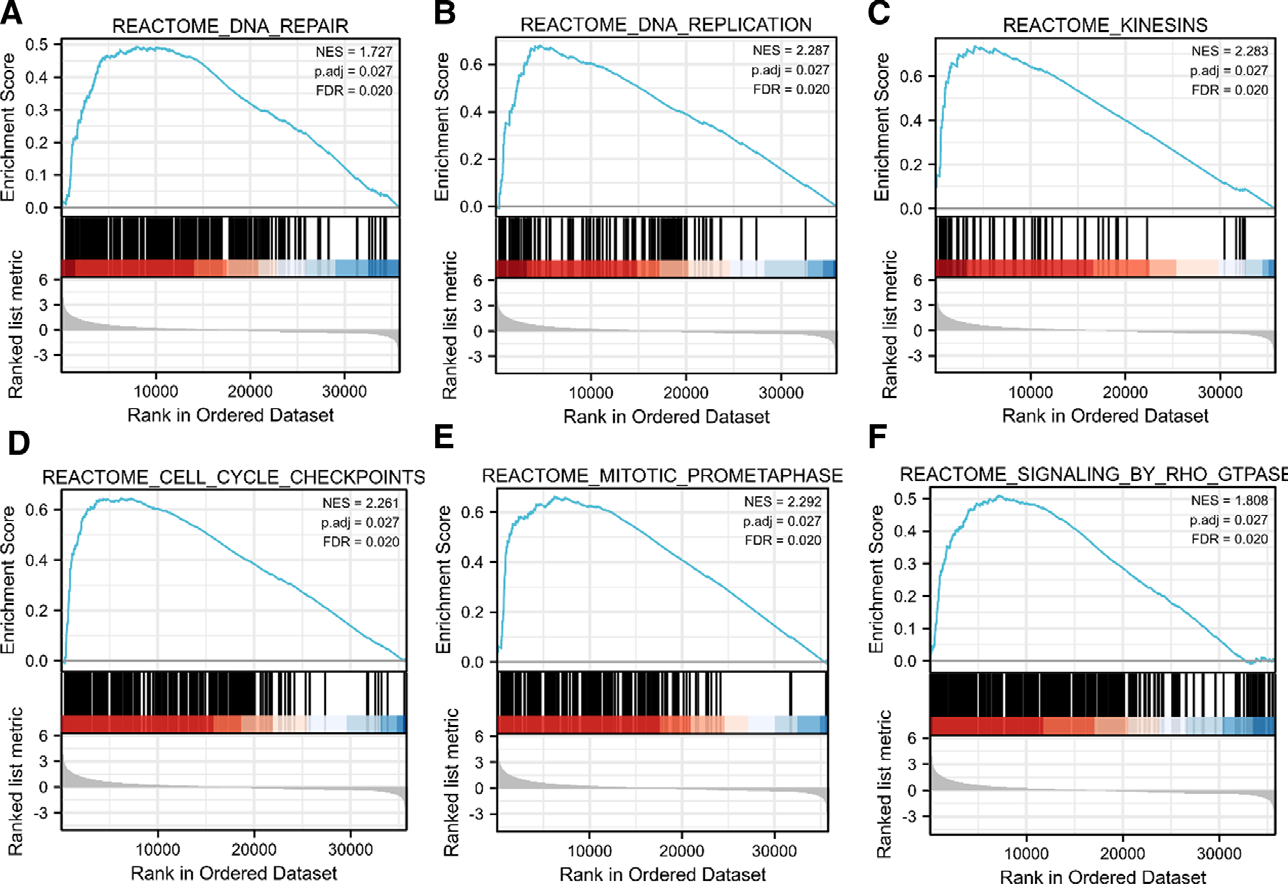
GSEA analyses in HCC patients with high expression of SKA1 compared with the ones with low expression. FDR = false discovery rate, GSEA = gene set enrichment analysis, HCC = hepatocellular carcinoma, NES = normalized enrichment score.

The underlying molecular processes of SKA1 in carcinogenesis and development were evaluated by functional enrichment analysis. The 73 molecules interacting with SKA1 were collected via the BioGRID online site, as shown in Figure 4A. In addition, we acquired the top 100 SKA1 co-expressed genes (Table S1, Supplemental Digital Content, http://links.lww.com/MD/J651) in HCC using GEPIA2.0. Among these, non-smc condensin i complex subunit g, ndc80 kinetochore complex component, cyclin A2, dlg-associated protein 5, exonuclease 1, and polo-like kinase 1 were highly correlated with SKA1 in HCC tissues (Fig. 4B and C). The effects of SKA1 co-expressed genes on the control of the cell cycle and oocyte meiosis in cancer and development were revealed by GO and KEGG enrichment analyses (Fig. 4D). Next, we used GSEA to compare the signaling pathways between SKA1 datasets with low and high expression levels (Fig. 5). We discovered that SKA1 is associated with kinesins, DNA replication, DNA repair, Rho GTPase signaling, mitotic prometaphase, and cell cycle checkpoints.

### 3.4. Associations between SKA1 expression and multiple clinicopathological

#### 3.4.1. Characteristics in hepatocellular carcinoma.

The clinical data of 374 HCC patients included age, sex, T stage, N stage, M stage, pathologic stage, histologic grade, race, weight, tumor status, OS event, DSS event, PFI event, age, AFP (ng/mL), and prothrombin time (Table 1). Patients with HCC with a mean age of 64 years were included in the present study. The chi-square test showed that SKA1 expression was significantly correlated with T stage (*P* = .002), pathologic stage (*P* < .001), tumor status (*P* = .016), weight (*P* = .005), histologic grade (*P* < .001), prothrombin time (*P* = .032), OS event (*P* = .013), DSS event (*P* = .011), and PFI event (*P* = .039). SKA1 was substantially associated with histologic grade according to Fisher’s exact test (*P* < .001). The Wilcoxon rank-sum test showed that SKA1 was significantly correlated with age (*P* = .026) and AFP (ng/mL) (*P* < .001).

**Table 1:** Demographic and clinicopathological parameters of HCC patients in TCGA–LIHC.

Characteristic	Low expression of SKA1	High expression of SKA1	*P*
Total number of patients	187	187	
T stage, n (%)			
T1	109 (29.4%)	74 (19.9%)	.002
T2	37 (10%)	58 (15.6%)	
T3	33 (8.9%)	47 (12.7%)	
T4	5 (1.3%)	8 (2.2%)	
N stage, n (%)			
N0	124 (48.1%)	130 (50.4%)	.623
N1	1 (0.4%)	3 (1.2%)	
M stage, n (%)			
M0	129 (47.4%)	139 (51.1%)	.056
M1	4 (1.5%)	0 (0%)	
Pathologic stage, n (%)			
Stage I	102 (29.1%)	71 (20.3%)	<.001
Stage II	35 (10%)	52 (14.9%)	
Stage III	32 (9.1%)	53 (15.1%)	
Stage IV	5 (1.4%)	0 (0%)	
Tumor status, n (%)			
Tumor free	113 (31.8%)	89 (25.1%)	.016
With tumor	65 (18.3%)	88 (24.8%)	
Race, n (%)			
Asian	68 (18.8%)	92 (25.4%)	.064
Black or African American	8 (2.2%)	9 (2.5%)	
White	102 (28.2%)	83 (22.9%)	
Weight, n (%)			
≤70	79 (22.8%)	105 (30.3%)	.005
>70	95 (27.5%)	67 (19.4%)	
Histologic grade, n (%)			
G1	39 (10.6%)	16 (4.3%)	<.001
G2	99 (26.8%)	79 (21.4%)	
G3	45 (12.2%)	79 (21.4%)	
G4	2 (0.5%)	10 (2.7%)	
Prothrombin time, n (%)			
≤4	101 (34%)	107 (36%)	.032
>4	56 (18.9%)	33 (11.1%)	
OS event, n (%)			
Alive	134 (35.8%)	110 (29.4%)	.013
Dead	53 (14.2%)	77 (20.6%)	
DSS event, n (%)			
Alive	154 (42.1%)	133 (36.3%)	.011
Dead	29 (7.9%)	50 (13.7%)	
PFI event, n (%)			
Alive	106 (28.3%)	85 (22.7%)	.039
Dead	81 (21.7%)	102 (27.3%)	
Age, median (IQR)	64 (53.5, 70)	59 (51, 67.75)	.026
AFP (ng/mL), median (IQR)	6 (3, 37.5)	50.5 (9, 1744.75)	<.001

**Figure 6.**
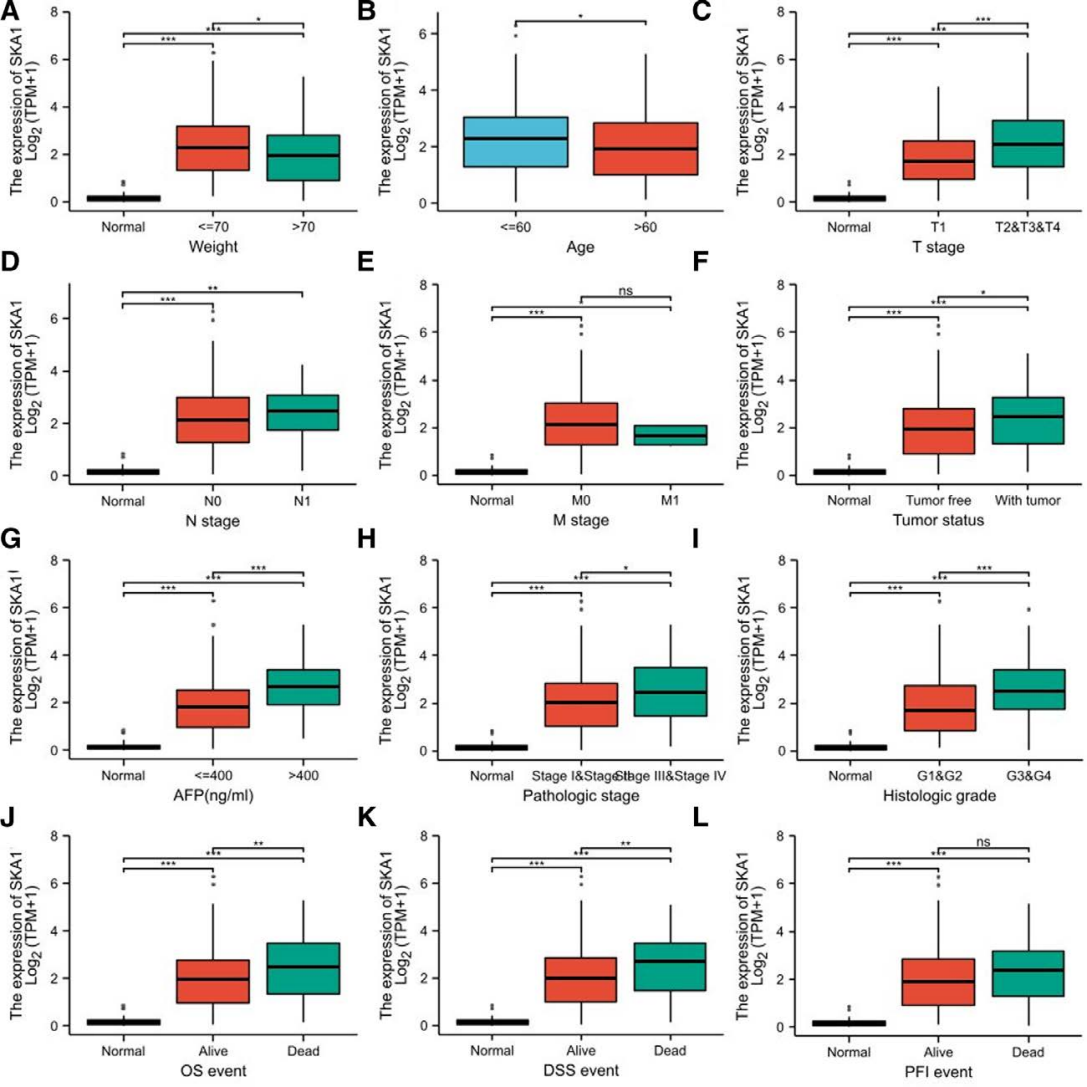
SKA1 expression levels in HCC patients correspond with a variety of clinicopathological features. (A–L) The correlation analysis between SKA1 expression levels and (A) weight, (B) age, (C) T stages, (D) N stages, (E) M stages, (F) tumor status, (G) AFP levels, (H) pathologic stages, (I) histologic grade, (J) OS, (K) DSS, and (L) PFI of HCC patients. *P < .05, **P < .01, ***P < .001, ^ns^*P* > .05. AFP = alpha-fetoprotein, DSS = disease-specific survival, HCC = hepatocellular carcinoma, OS = overall survival, PFI = progression-free interval.

**Figure 7.**
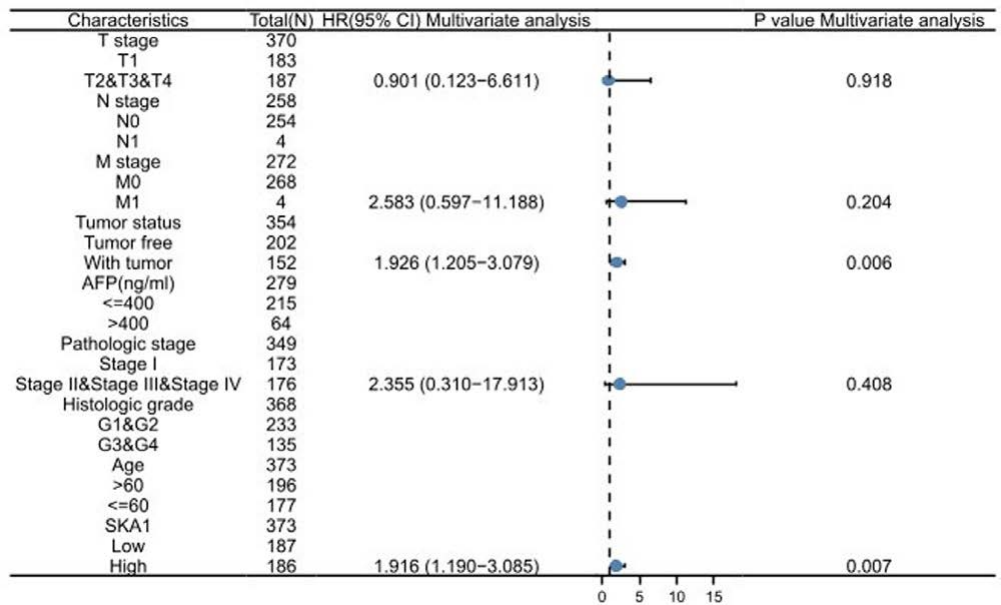
Multivariate Cox regression visualized in the forest plot.

SKA1 expression levels were significantly correlated with weight (Fig. 6A), age (Fig. 6B), T stage (Fig. 6C), N stage (Fig. 6D), M stage (Fig. 6E), tumor status (Fig. 6F), AFP levels (Fig. 6G), pathologic stages (Fig. 6H), histologic grade (Fig. 6I), OS (Fig. 6J), DSS (Fig. 6K), and PFI (Fig. 6L) of the HCC patients. SKA1 expression was significantly higher in patients with HCC aged < 60 years than in those aged > 60 years. In addition, HCC patients with high AFP levels, low OS, low PFI, low DSS rates, and advanced disease stages had increased SKA1 levels. Logistic regression analysis revealed that SKA1 expression levels in HCC tissues were strongly linked to T stage, tumor status, histologic grade, age, and AFP levels (Table 2). In the Cox regression model, univariate Cox regression indicates that the T stage (*P* < .001), M stage (*P* = .017), tumor status (*P* < .001), pathologic stage (*P* < .001), and SKA1 (*P* = .003) were correlated with a bad prognosis of HCC (Table 3). Multivariate Cox regression analysis showed that tumor status (*P* = .006) and SKA1 (*P* = .007) were independent prognostic factors for OS (Fig. 7).

**Table 2:** Logistic regression analysis of the relationship between clinicopathological characteristics and the SKA1 expression levels in HCC patients.

Characteristics	Total (N)	Odds ratio	*P* value
T stage (T2&T3&T4 vs T1)	371	2.219 (1.469–3.372)	<.001
N stage (N1 vs N0)	258	2.862 (0.361–58.271)	.365
Pathologic stage (Stage III & Stage IV vs Stage I & Stage II)	350	1.595 (0.985–2.606)	.059
Tumor status (With tumor vs Tumor free)	355	1.719 (1.127–2.634)	.012
Histologic grade (G3&G4 vs G1&G2)	369	2.751 (1.780–4.293)	<.001
Age (>60 vs ≤60)	373	0.629 (0.417–0.946)	.026
AFP (ng/ml) (>400 vs ≤400)	280	3.185 (1.783–5.860)	<.001

**Table 3:** Univariate and multivariate analyses of clinical pathological parameters in HCC patients.

Characteristics	Total (N)	Univariate analysis		Multivariate analysis	
		Hazard ratio (95% CI)	P value	Hazard ratio (95% CI)	P value
T stage	370				
T1	183	Reference			
T2&T3&T4	187	2.126 (1.481–3.052)	**<.001**	0.901 (0.123–6.611)	.918
N stage	258				
N0	254	Reference			
N1	4	2.029 (0.497–8.281)	.324		
M stage	272				
M0	268	Reference			
M1	4	4.077 (1.281–12.973)	**.017**	2.583 (0.597–11.188)	.204
Tumor status	354				
Tumor free	202	Reference			
With tumor	152	2.317 (1.590–3.376)	**<.001**	1.926 (1.205–3.079)	**.006**
AFP (ng/ml)	279				
≤400	215	Reference			
>400	64	1.075 (0.658–1.759)	.772		
Pathologic stage	349				
Stage I	173	Reference			
Stage II & Stage III & Stage IV	176	2.090 (1.429–3.055)	**<.001**	2.355 (0.310–17.913)	.408
Histologic grade	368				
G1&G2	233	Reference			
G3&G4	135	1.091 (0.761–1.564)	.636		
Age	373				
>60	196	Reference			
≤60	177	0.830 (0.585–1.176)	.295		
SKA1	373				
Low	187	Reference			
High	186	1.693 (1.192–2.403)	**.003**	1.916 (1.190–3.085)	**.007**

*P* <.05, and the results were statistically significant.

### 3.5. Subgroup analyses to test the prognostic capability of SKA1 in HCC

**Figure 8.**
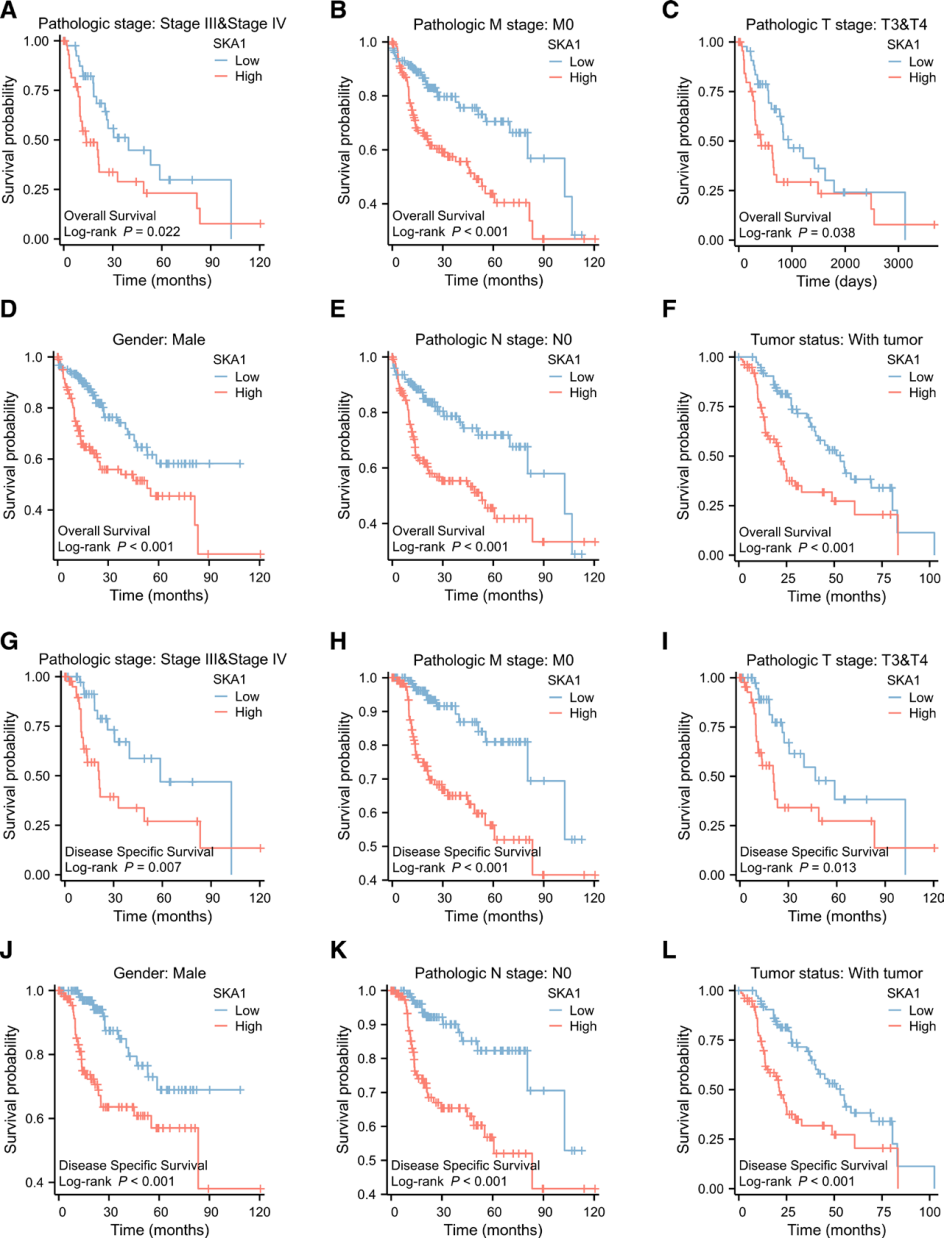
Kaplan–Meier curves for OS and DSS in HCC. (A–F) K–M curves for OS in different subgroups. (G–L) K–M curves for DSS in different subgroups. DSS = disease-specific survival, HCC = hepatocellular carcinoma, OS = overall survival, K–M = Kaplan–Meier.

Kaplan–Meier survival analysis revealed that increased SKA1 expression was associated with poor prognosis (*P* = .003). To further investigate the predictive power of SKA1 for HCC, subgroup analyses were conducted. Patients were stratified based on several clinicopathological factors, including pathologic stage, M stage, sex, T stage, sex, N stage, sex, and tumor status. Subgroup analysis by different clinical features revealed that high SKA1 expression was strongly associated with poor prognosis in LIHC cases stage III and IV LIHC (OS: *P =* .022, DSS: *P =* .007), M0 (OS and DSS: *P* < .001), T3 and T4 (OS: *P =* .038, DSS: *P =* .013), male (OS and DSS: *P* < .001), N0 (OS and DSS: *P* < .001), with tumor (OS and DSS: *P <* .001), as shown in Figure 8A–L.

### 3.6. Construction and validation of a nomogram based on the independent factors

**Figure 9.**
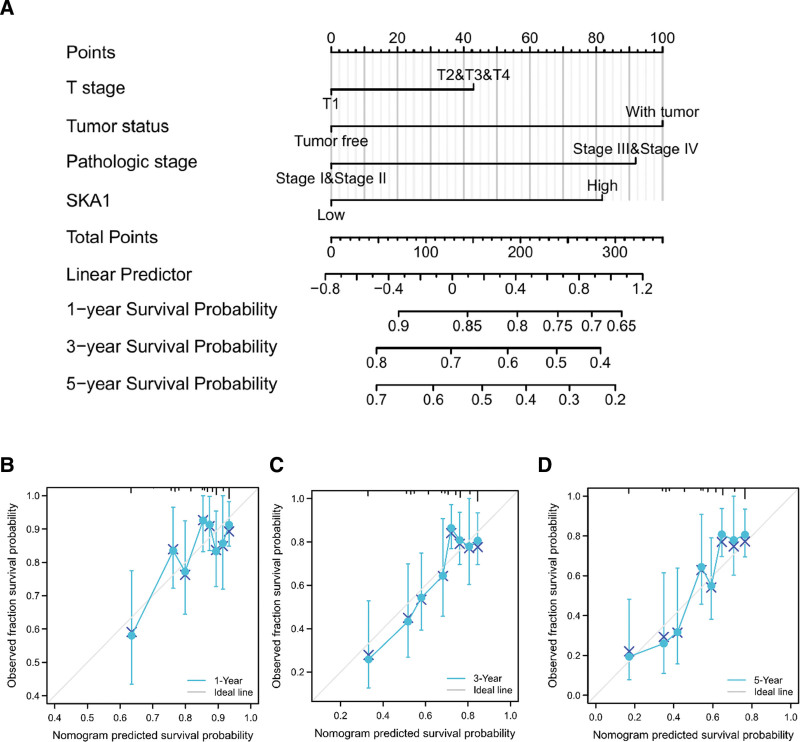
A nomogram with calibration curves for estimating the 1, 3, and 5-yr OS rates of HCC patients. (A) A nomogram for prediction of 1, 3, and 5-yr OS rates of patients with HCC. (B–D) Calibration curves of the nomogram prediction of 1, 3, and 5-yr OS rates of patients with HCC. HCC = hepatocellular carcinoma, OS = overall survival.

A nomogram based on independent factors of OS was created to predict the prognosis of patients with HCC. A higher total nomogram score was associated with poorer prognosis (Fig. 9A). In addition, calibration curves were employed to evaluate the predictive efficacy of the nomogram (Fig. 9B–D).

### 3.7. Relationship between methylation and expression of SKA1

**Figure 10.**
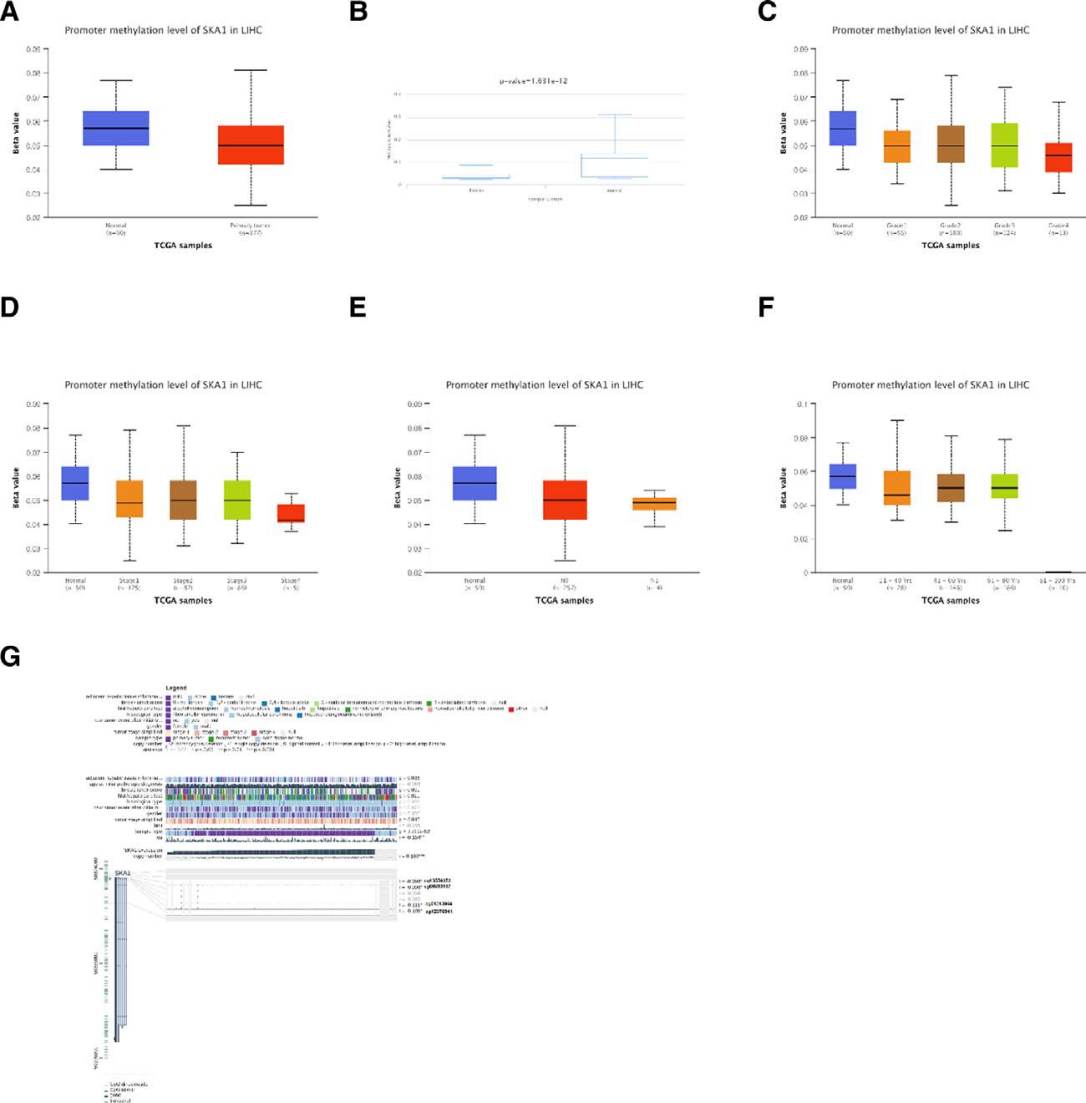
SKA1 DNA methylation levels and their predictive significance for HCC. (B) DiseaseMeth 2.0 was used to evaluate methylation. (C–F) The promoter methylation level of SKA1 in HCC tissues of various tumor grade (C), tumor stage (D), N stage (E), and (F) age by the UALCAN database. (G) MEXPRESS was utilized to visualize the methylation location of the SKA1 DNA sequence correlation with gene expression. The blue line in the middle of the plot serves as an example of SKA1 expression. The right side displays the methylation locations and query gene expression Pearson’s correlation coefficients and *P* values. HCC = hepatocellular carcinoma.

UALCAN research revealed that SKA1 promoter methylation levels were considerably lower in HCC tissues than in normal samples (Fig. 10A). Similarly, DiseaseMeth version 2.0 analysis showed that the methylation of SKA1 was considerably lower in HCC tissues than in paracancerous normal tissues (Fig. 10B). The methylation status of SKA1 was high in low-grade tumors, early stage tumors, N0 stage tumors, and middle-aged and elderly patients (Fig. 10C–F). Additionally, we identified 4 methylation sites in the DNA sequences of SKA1 (cg13556235, cg06893537, cg01313966, and cg12376941) that were adversely associated with their expression levels (Fig. 10G).

### 3.8. Construction of mRNA and miRNA co-expression network and related miRNA prognostic survival curve analysis

**Figure 11.**
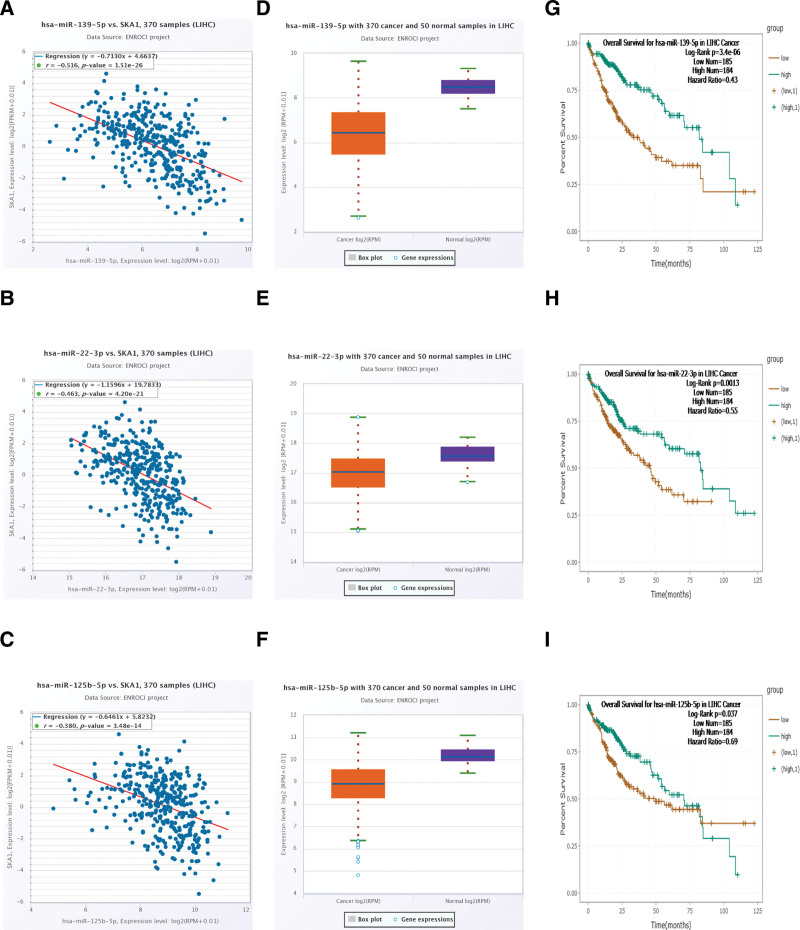
Correlation analysis between SKA1 and miR-139-5p, miR-22-3p, miR-125b-5p, differential expression, and survival curve analyses for miR-139-5p, miR-22-3p, miR-125b-5p in HCC. (A–C) Correlation between SKA1 and 3 miRNAs. (D–F) differential expression of 3 miRNAs in HCC. (G–I) K–M plot for each miRNA in HCC. HCC = hepatocellular carcinoma, K–M = Kaplan–Meier.

We retrieved SKA1-miRNA interaction data from the starBase database and performed an analysis to find relevant microRNA (miRNA)-messageRNA (mRNA) interaction networks associated with HCC prognosis. SKA1 was shown to have a negative connection with the 3 categories of miRNAs (*R* value < –0.3, and *P* value < .001, Fig. 11A–C). Finally, to test the possible targets and pathways, we created an mRNA-miRNA co-expression network. were considerably overexpressed in the tumor group (*P < .001*, Fig. 11D–F), and the above-mentioned linked 3 miRNAs K–M curve revealed that the high-expression group outperformed the low-expression group in terms of prognosis (*P < .05*, Fig. 11G–I).

### 3.9. Establishment of mRNA-miRNA-lncRNA interaction network and prognostic survival analysis of SKA1-related miRNA and lncRNA

**Figure 12.**
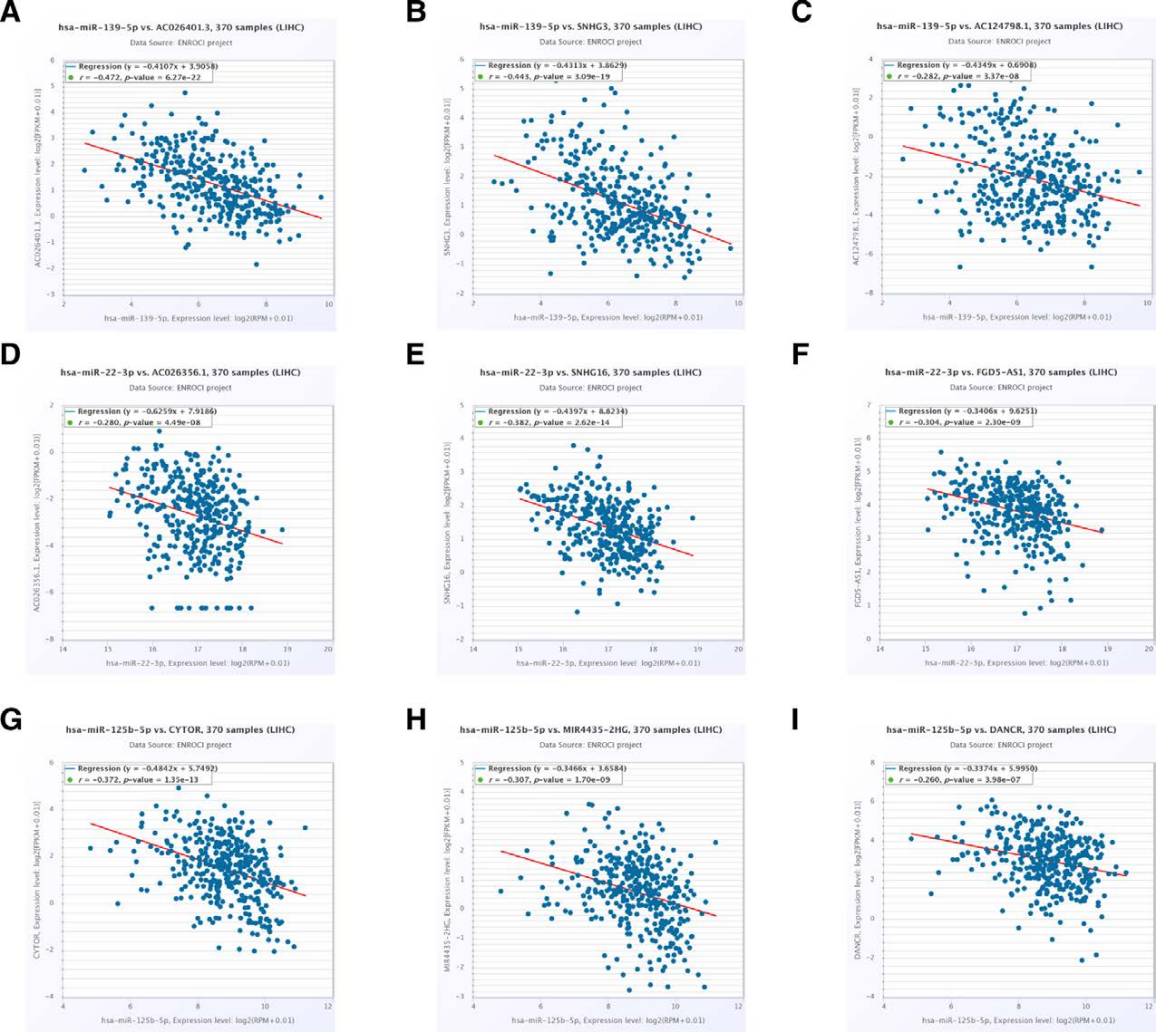
Correlation analysis between miRNA-SKA1-related lncRNA and miRNA. (A–C) Correlation analysis between miR-139-5p-related lncRNA and miR-139-5p. (D–F) Correlation analysis between miR-22-3p-related lncRNA and miR-22-3p. (G–I) Correlation analysis between miR-125b-5p-related lncRNA and miR-125b-5p.

**Figure 13.**
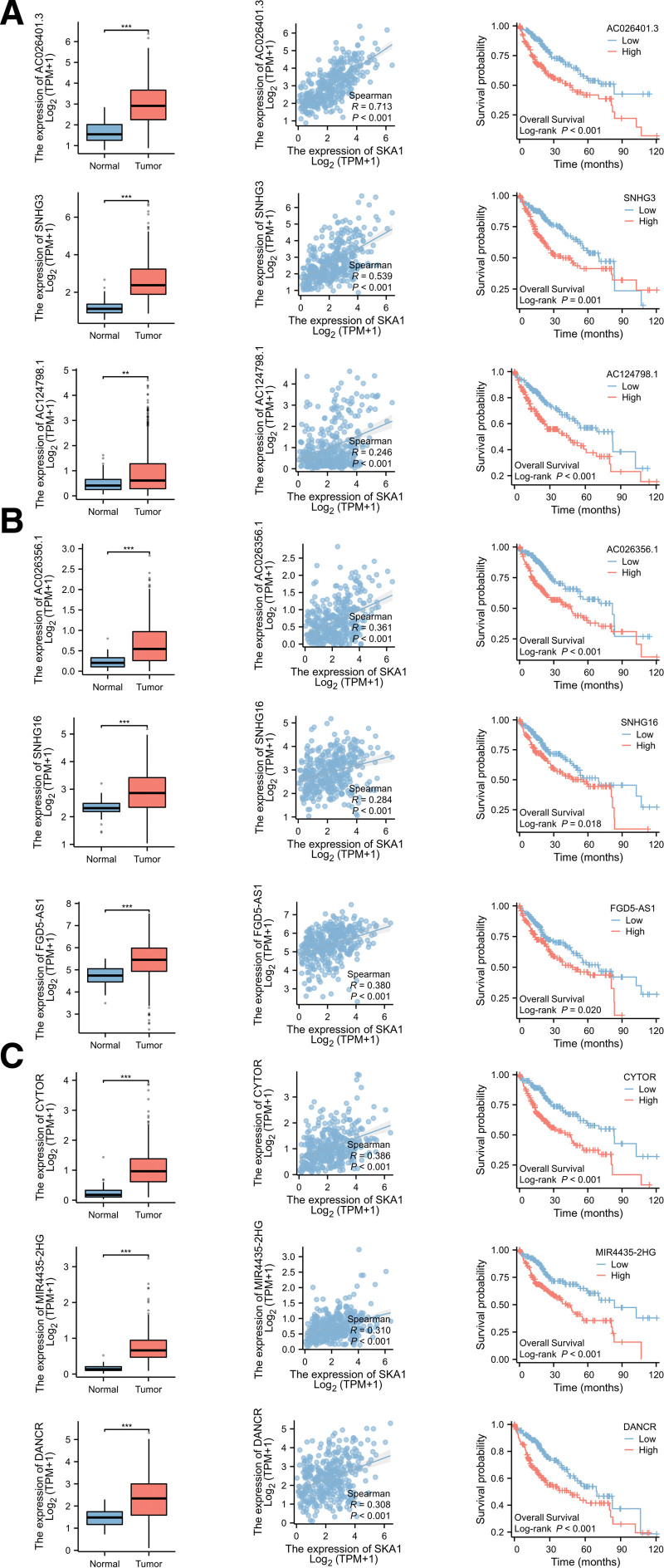
Correlation, difference, and survival curve analyses between miRNA-SKA1-related lncRNA and SKA1, respectively. (A) Correlation, difference, and survival curve analyses between miR-139-5p-SKA1-related lncRNA and SKA1, respectively. (B) Correlation, difference, and survival curve analyses were performed on miR-22-3p-SKA1-related lncRNA and SKA1. (C) Correlation, difference, and survival curve analyses between miR-125b-5p-SKA1-related lncRNA and SKA1, respectively.

**Figure 14.**
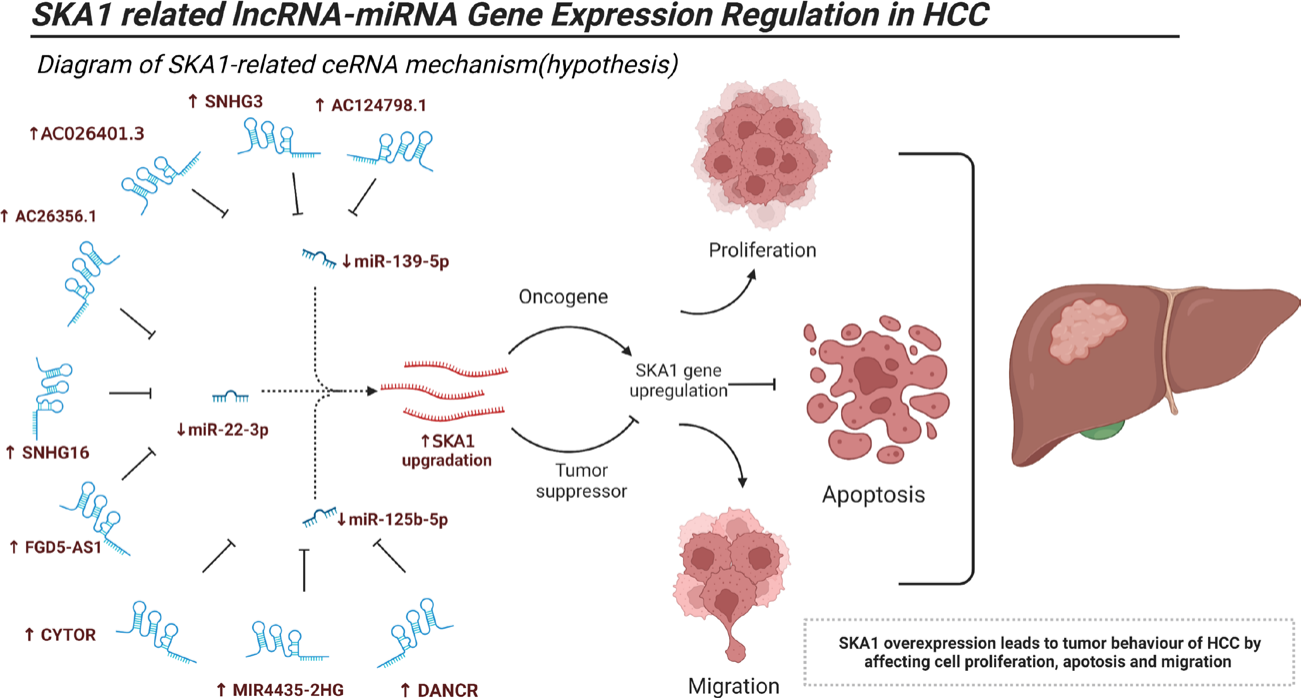
Conceptual map diagram of SKA1-related ceRNA hypothesis in HCC. HCC = hepatocellular carcinoma.

Furthermore, we retrieved lncRNA data that interacted with miR-139-5p, miR-22-3p, and miR-125b-5p from the starBase database. Then, using the R programming language, we sorted and evaluated the correlation coefficients (*R* value > 0.2), log FC values (log FC value > 0), survival curves, and differential expression between the tumor group and normal group (*P* < .01). In addition, we chose lncRNAs whose expression levels were positively linked with SKA1 according to the data (those with *R* value *>* 0.2, and *P* value < .001). Eventually, miR-139-5p-related lncRNAs (i.e., AC026401.3, SNHG3, and AC124798.1), miR-22-3p-related lncRNAs (i.e., AC26356.1, Small Nucleolar RNA Host Gene 16 (SNHG16), and FGD5 Antisense RNA 1(FGD5-AS1)), and miR-125b-5p-related lncRNAs (i.e., Cytoskeleton Regulator RNA (CYTOR), MIR4435-2 host gene, and Differentiation Antagonizing Non-Protein Coding RNA(DANCR)) were identified (Figs. 12A–I and 13A–C. Based on these findings, a potential ceRNA mechanism for SKA1 is shown (Fig. 14).

### 3.10. Correlation analyses of SKA1 with various immune cells and several immune checkpoints

**Figure 15.**
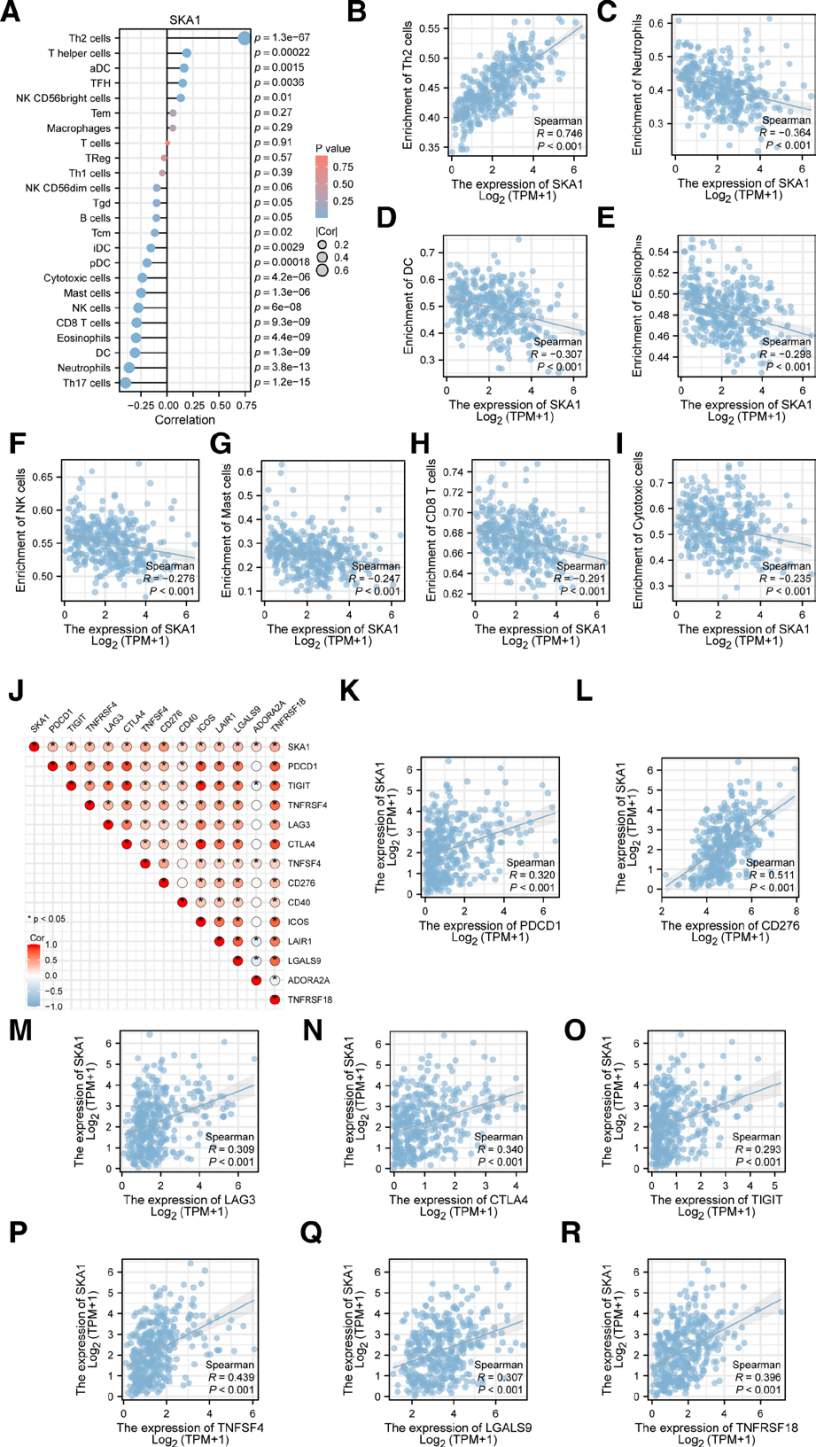
Correlations between SKA1 and several immune cells and immune checkpoints molecule.

Furthermore, we investigated the relationships between immune cells and the degree of immune infiltration of SKA1 and discovered that the Th2 cell cluster was positively linked with SKA1 (*R* > 0.2, *P <* .001), while Th17 cells, Neutrophils, Dendritic cells (DC), Eosinophils, CD8 T cells, natural killer cells, mast cells, and cytotoxic cells were negatively correlated with E2F Transcription Factor 1 (*R*<–0.2, *P* < .001; Fig. 15A–I). We also conducted correlation analysis between SKA1 expression and several immunological checkpoints. TCGA-LIHC database-related immune checkpoint research revealed that SKA1 expression was correlated positively with Programmed Cell Death 1, CD276 molecule, Lymphocyte Activating 3, Cytotoxic T-Lymphocyte Associated Protein 4, T cell immunoreceptor with immunoglobulin and ITIM domains, TNF Superfamily Member 4, galectin 9, and TNF Receptor Superfamily Member 18 (*R* > 0.2, *P* < .001; Fig. 15J–R).

## 4. Discussion

HCC is one of the deadliest cancers in the world and has a poor prognosis despite improved early diagnosis and combined therapy.^[26]^ Currently, serum AFP, ultrasonography, and CT scanning are important methods for the early diagnosis of HCC; however, the misdiagnosis rate is high.^[27,28]^ Therefore, the investigation of effective prognostic biomarkers is a pivotal area among the several considerations in HCC research. SKA1 is a member of the SKA complex, which includes SKA1, spindle, kinetochore-associated complex subunit 2 (SKA2), and spindle and kinetochore-associated complex subunit 3 (SKA3). SKA complexes bind to microtubules and form oligomeric components.^[29]^ Spindle and kinetochore-associated complex subunit 1 (SKA1), a newly discovered gene associated with mitosis,^[30]^ has been found to silence the spindle checkpoint.^[14]^ SKA1 is a subtype of the SKA complex that causes spindle microtubules to firmly attach to the kinetochore during mitosis.^[14,31]^ In liver cancer, SKA1 silencing significantly inhibits liver cancer cell colony formation and leads to S phase cell cycle arrest, indicating that SKA1 may be associated with cancer cell proliferation and is involved in the tumorigenesis of hepatocellular carcinoma.^[32]^

In this study, we investigated the expression, ceRNA network, and diagnostic and prognostic accuracies of SKA1 in HCC. We utilized bioinformatics analysis of sequencing data from TCGA to gain a comprehensive understanding of the potential role of SKA1 in HCC and provide directions for future HCC research.

Elevated SKA1 expression in HCC is associated with advanced clinicopathological features (AFP, histological grade, pathological stage, T stage, and tumor status), poor prognosis, and survival time. Furthermore, in univariate and multivariate Cox regression analyses, we found that after removing confounding factors, SKA1 remained an independent prognostic factor with a higher prognostic value than many other clinical variables, including AFP. Our results suggested that SKA1 is a potential prognostic and diagnostic marker that warrants further clinical validation. The function of SKA1 in HCC was further investigated by GSEA using the TCGA data.

Protein–protein interaction networks indicated that SKA1 can interact with the SPC24 Component Of NDC80 Kinetochore Complex, Nudix Hydrolase 5, SNAP-Associated Protein, Coiled-Coil Alpha-Helical Rod Protein 1, KIAA0753, SKA2, MIS12 Kinetochore Complex Component (MIS12), Kinesin Family Member 2A, and Synaptosome Associated Protein 29 (SNAP29), in addition to the subunit protein of Protein Phosphatase 2 regulatory subunit beta. The Protein Phosphatase 2 regulatory subunit beta subfamily of serine/threonine phosphatases is involved in various cellular processes including cell cycle regulation, signal transduction, and transcriptional regulation. They are also involved in the regulation of actin cytoskeleton dynamics, cell adhesion, and cell migration.^[33]^ GSEA showed that cell cycle checkpoints, DNA replication, DNA repair, Rho GTPase signaling, mitotic prometaphase, and kinesins in HCC were enriched in the SKA1 overexpression phenotype. These findings indicate that SKA1 may participate in the regulation of cell cycle and cancer-promoting pathways in HCC.

DNA methylation is a common epigenetic mechanism of gene regulation that generally silences gene expression.^[34]^ Our study showed that SKA1 tended to have higher methylation levels in normal liver tissues than in HCC tissues. Additionally, we further investigated the underlying mechanism of SKA1 overexpression in Hepatocellular Carcinoma, and our data showed that SKA1 overexpression may be related to its DNA hypomethylation. SKA1 hypomethylation is associated with poor prognosis in patients with HCC.

MiR-139-5p is thought to be a cancer suppressor because it is downregulated in several types of cancers such as glioma,^[35]^ colorectal cancer,^[36,37]^ gastric cancer,^[38]^ and bladder cancer.^[39]^ In gastric cancer, miR-139-5p may inhibit tumor cell proliferation by negatively regulating Peripheral Myelin Protein 22 by targeting the NF-κB signal pathway.^[40]^ MiR-22-3p plays an important role in suppressing tumor progression in many cancers.^[41–43]^ It has been confirmed that miR-22-3p can inhibit osteosarcoma progression by targeting and regulating Transcription Factor 7 Like 2.^[44]^ Low miR-125-5p expression is associated with the progression of various tumors.^[45–47]^ In addition, miR-125-5p inhibits the proliferation and invasion of breast cancer cells by targeting and regulating Tumor Protein D52.^[48]^ Previous studies have shown that overexpression of lncRNA SNHG3 is associated with poor prognosis in hepatocellular carcinoma.^[49]^ LncRNA SNHG3 may mediate the malignant proliferation of hepatocellular carcinoma by regulating Nei Like DNA Glycosylase 3 via transcription factor E2F Transcription Factor 1.^[50]^ AC026401.3 is upregulated in hepatocellular carcinoma tissues. Notably, AC026401.3 may enhance resistance to sorafenib by interacting with OCT1 and activating the E2F Transcription Factor 2 signaling pathway in hepatocellular carcinoma.^[51]^ High SNHG16 expression is associated with postoperative tumor recurrence and poor prognosis in HCC. SNHG16 promotes HCC progression of hepatocellular carcinoma by activating the ECM receptor interaction pathway.^[52]^ FGD5-AS1 is upregulated in hepatocellular carcinoma, predicts poor prognosis, and promotes the proliferation of hepatocellular carcinoma cells by targeting miR-223 and regulating the expression of Epithelial Cell Transforming 2 and FAT Atypical Cadherin 1.^[53]^ Previous studies have shown that CYTOR has the potential to be a prognostic biomarker for HCC,^[54]^ and it can promote the progression of hepatocellular carcinoma by targeting the miR-125a-5p/LIM And SH3 Protein 1 axis.^[55]^ DANCR is upregulated in hepatocellular carcinoma cells and plays a role in promoting the occurrence and progression of hepatocellular carcinoma. Knockout of DANCR can delay the progression and initiation of hepatocellular carcinoma in an in situ hepatoma mouse model of patient-derived xenograft.^[56]^ MIR4435-2 host gene has previously been reported as an oncogene in hepatocellular carcinoma and can promote the proliferation of cancer cells by upregulating miRNA-487a and UDP-GlcNAc:BetaGal Beta-1,3-N-Acetylglucosaminyltransferase 5.^[57,58]^

In a previous study, approximately 25% of HCC samples showed expression of biomarkers for inflammatory responses.^[59]^ Our study demonstrated a potential relationship between SKA1 expression and tumor immune cell infiltration. SKA1 expression was negatively correlated with the number of neutrophils, DC, and CD8 T cells in HCC tissues. Neutrophils can directly kill cancer cells by secreting cytotoxic substances, such as ROS, nitric oxide, and neutrophil elastase.^[60]^ DC-mediated cross-priming of tumor-specific CD8^+^ T cells plays a critical role in initiating and sustaining antitumor immunity.^[61–64]^ Our data showed a positive correlation between SKA1 expression and the proportion of type 2 T helper cells, T follicular helper cells, and T helper cells in HCC tissues. T follicular helper cells are characterized by the expression of the C-X-C motif chemokine receptor 5 (CXCR5) membrane marker and the receptor of chemokine (C-X-C motif) ligand 13 (CXCL13), which are required for their migration to the germinal center.^[65,66]^ CXCL13/CXCR5 plays a major role in cancer cell growth. A shift in the balance between type 1 and type 2 T helper cells (Th1 and Th2 cells) is considered an important factor in cancer development.^[67,68]^ Furthermore, Th2-derived cytokines such as interleukin-4 and interleukin-13 promote tumor progression by inducing M2 macrophage polarization.^[69]^ Therefore, our data suggested that SKA1 overexpression plays a significant role in the immune escape mechanisms of HCC cells, thereby contributing to HCC growth and progression.

CTLA-4 and PDCD-1 are 2 critical proteins associated with tumor immune escape.^[70,71]^ Immune checkpoint inhibitors, such as ipilimumab (CTLA-4 inhibitor) and nivolumab (PDCD-1 inhibitor), significantly improve the overall survival rates of patients with melanoma^[72,73]^ and advanced liver cancer.^[74]^ Therefore, we analyzed the association between SKA1 expression and immunological checkpoint genes, including CTLA-4 and PDCD-1. In HCC tissues, positive correlations were observed between the expression levels of SKA1 and the expression levels of immunological checkpoint genes. These results indicate that SKA1 could be a target for immunotherapy in patients with HCC.

This study revealed that SKA1 is a biomarker associated with HCC prognosis. SKA1 expression and methylation status of SKA1 not only correlated with immune cell infiltration, but also with immune checkpoint genes. We also been used SKA1-related lncRNAs to create ceRNA models. It should be emphasized that this study relied on bioinformatics analysis; therefore, the accuracy of the findings must be confirmed through additional exploratory studies. However, our results provide a preferred choice and reference for future studies on the transcription factor SKA1, which may be useful in discovering research targets for future molecular therapies and immunotherapies for HCC.

## 5. Conclusion

This study demonstrates the diagnostic and prognostic value of SKA1 in HCC. SKA1 affects HCC growth by controlling the expression of genes involved in DNA replication and chromosome separation. Methylation and gene expression of SKA1 were related to HCC prognosis. SKA1 expression levels correspond to the degree of tumor infiltration by various immune cell types, and may play a role in the therapeutic response of patients with HCC. Bioinformatics analysis led us to use the ceRNA mechanism as a starting point for analyzing SKA1-related lncRNAs and miRNAs. Therefore, SKA1 is a viable therapeutic target and valuable diagnostic and prognostic biomarker for HCC. However, additional studies are required to confirm these findings.

## Acknowledgements

We acknowledge the Sun Yat-sen University Library for providing the technical support.

## Author contributions

**Conceptualization:** Zhiqi Xu, Fanjing Zeng.

**Data curation:** Zhiqi Xu.

**Formal analysis:** Zhiqi Xu, Fanjing Zeng, Peng Zhuang.

**Funding acquisition:** Peng Zhuang.

**Investigation:** Fanjing Zeng.

**Methodology:** Zhiqi Xu, Fanjing Zeng.

**Project administration:** Peng Zhuang.

**Resources:** Peng Zhuang.

**Software:** Zhiqi Xu, Fanjing Zeng.

**Supervision:** Peng Zhuang.

**Validation:** Fanjing Zeng.

**Visualization:** Zhiqi Xu, Fanjing Zeng.

**Writing – original draft:** Zhiqi Xu, Fanjing Zeng.

**Writing – review & editing:** Peng Zhuang.

## Supplementary Material


